# Mechanically Strong and Flame-Retardant Cellulose-Based Aerogel Prepared via Phosphorylation-Coupled Ca^2+^ Coordination

**DOI:** 10.3390/gels11060408

**Published:** 2025-05-29

**Authors:** Yadong Zhao, Chengcheng Peng, Zheng Yang, Zhengjie Liu, Heng Yen Khong, Soottawat Benjakul, Bin Zhang, Ruizhi Yang

**Affiliations:** 1 School of Food and Pharmacy, Zhejiang Ocean University, Zhoushan 316022, China; zhaoyd@zjou.edu.cn (Y.Z.); qj18156421716@163.com (C.P.); yangzheng@zjou.edu.cn (Z.Y.); zhangbin@zjou.edu.cn (B.Z.); 2 Faculty of Applied Sciences, Universiti Teknologi MARA, Kota Samarahan 94300, Sarawak, Malaysia; khonghy@uitm.edu.my; 3 International Center of Excellence in Seafood Science and Innovation (ICE-SSI), Faculty of Agro-Industry, Prince of Songkla University, Hat Yai 90110, Songkhla, Thailand; soottawat.b@psu.ac.th

**Keywords:** nanocellulose, aerogel, flame retardant, phosphorylation, Ca^2+^ coordination

## Abstract

Cellulose-based aerogel is an environmentally friendly multifunctional material that is renewable, biodegradable, and easily surface-modified. However, due to its flammability, cellulose serves as an ignition source in fire incidents, leading to the combustion of building materials and resulting in significant economic losses and safety risks. Consequently, it is essential to develop cellulose-based building materials with flame-retardant properties. Initially, a porous cellulose-based flame-retardant aerogel was successfully synthesized through freeze-drying, utilizing lignocellulose as the raw material. Subsequently, phosphorylation of cellulose was coupled with Ca^2+^ cross-linking via self-assembly and surface deposition effects to enhance its flame-retardant properties. Finally, the synthesized materials were characterized using infrared spectroscopy, X-ray diffraction, thermogravimetric analysis, mechanical compression testing, and scanning electron microscopy. The aerogel of the phosphorylated cellulose nanofibrils cross-linked via 1.5% CaCl_2_ exhibited the most effective flame-retardant properties and the best mechanical characteristics, achieving a UL-94 test rating of V-0 and a maximum flame-retardant rate of 90.6%. Additionally, its compressive strength and elastic modulus were recorded at 0.39 and 0.98 MPa, respectively. The preparation process is environmentally friendly, yielding products that demonstrate significant flame-retardant effects and are non-toxic. This product is anticipated to replace polymer-based commercial aerogel materials, representing a sustainable solution to the issue of “white pollution”.

## 1. Introduction

In recent years, the increasing scarcity of petroleum resources, coupled with the significant environmental pollution associated with petroleum-based polymers, has prompted researchers to explore biodegradable, green, and environmentally friendly natural polymer materials [1]. Cellulose—the most abundant natural polymer—serves as a renewable organic resource [2], whose chemical structure consists of a linear polymer made up of multiple β-D-pyran glucose units linked via 1,4-β glycosidic bonds [3]. Cellulose molecules feature a variety of hydrogen bond systems that significantly influence their properties, including solubility, hydroxyl group reactivity, and crystallinity [4]. Each anhydroglucose unit (AGU) in the cellulose molecular chain contains three hydroxyl functional groups, leading to a substantial number of hydroxyl groups throughout cellulose molecules [5]. This abundance of hydroxyl groups enhances the excellent chemical modification capabilities of cellulose, facilitating various chemical reactions such as oxidation, etherification, and grafting [6]. Consequently, these modifications can improve the performance of cellulose and broaden its applications across diverse fields [7].

Cellulose-based aerogel materials exhibit low thermal conductivity, robust mechanical strength, and natural origin, making them valuable in the environmentally friendly green building industry [8]. However, cellulose materials are classified as organic, with a limited oxygen consumption value of approximately 19%, which categorizes them as flammable materials [9]. At present, the primary methods for improving the flame-retardant performance of cellulose materials include chemical modification and the application of flame retardants. For instance, Shi et al. (2017) grafted maleic anhydride onto cellulose and subsequently obtained cellulose fiber–Na^+^/Ca^2+^ through ion exchange with sodium and calcium salts [10]. Zhou et al. (2017) first dissolved aniline in a mixed acid of hydrochloric acid and phosphoric acid, followed by the in situ polymerization of bacterial cellulose with aniline [11]. Early research on fire retardants predominantly focused on halogen-based compounds. However, these halogen flame retardants release hydrogen halide gas, dioxins, and organic halides, which are not only toxic and corrosive but also pose a significant threat to human life and property safety. Moreover, these substances contribute to the depletion of the atmospheric ozone layer, leading to concerns regarding environmental protection and ecological sustainability [12]. As societal awareness of environmental issues has grown, the use of halogen flame retardants has become increasingly restricted. In contrast, inorganic flame retardants such as metal oxides and hydroxides exhibit poor washing resistance and require substantial quantities for effective application. This typically impacts the material formation process, as well as the physical and electrical properties of fibers, complicating their use. For example, when aluminum hydroxide is utilized for flame-retardant modification of viscose fibers, a complex coating process is necessary [13].

Consequently, there is an urgent need to develop an environmentally friendly, simple, and efficient method for promoting flame retardancy in the preparation of cellulose-based flame-retardant green building materials. Phosphorus flame retardants not only capture free radicals but also facilitate the formation of carbon residues, thereby achieving excellent flame-retardant properties. Furthermore, phosphorus-based flame retardants do not emit harmful substances upon combustion and have gained widespread usage in recent years [14]. Ye et al. (2025) explored a straightforward TDES pretreatment strategy that successfully introduced P/N/S groups onto the surfaces of lignin and holocellulose, resulting in the production of flame-retardant holocellulose (HC-SP) and lignin (lignin SP), respectively. The addition of phytic acid and sulfate significantly enhanced the thermal stability of lignin SP. By adjusting the ratio of these two components, nano-suspensions and nanocomposite films were prepared through a co-grinding process. The incorporation of lignin SP endowed the P/N/S-containing holocellulose nanofiber composite films with an excellent flame-retardant effect and considerable tensile strength [15].

This study aimed to develop a phosphorylated nanocellulose porous flame-retardant aerogel material characterized by high flame resistance and enhanced mechanical properties through a phosphorylation reaction combined with calcium ion cross-linking and freeze-drying molding technology. The specific research objectives were as follows: First, to synthesize the porous flame-retardant aerogel using Ca^2+^ cross-linking and freeze-drying technology, thereby creating an optimal material that reduces fire incidence rates and broadens the application of cellulose into new domains. Second, to conduct a structural analysis of the porous flame-retardant aerogel material with varying composition ratios utilizing techniques such as FTIR, XRD, and SEM. The results elucidate the structure–effect relationship between the microstructure of the porous flame-retardant material and its flame-retardant and mechanical properties. Furthermore, the mechanism underlying the effective flame-retardant effect of this innovative green material was analyzed, offering new insights into the production of high-performance flame-retardant green building materials.

## 2. Results and Discussion

### 2.1. Dimensions and Density of the Samples

The diameter, thickness, quality, density, and other characteristics of the samples prepared via freeze-drying using a vacuum freeze-dryer for 48 h are presented in Table 1. The data indicate that the density of the cellulose materials increased with the addition of Ca^2+^. However, it remained relatively low, with a density of only 0.110 ± 0.001 g/cm^3^ [16].

### 2.2. FTIR Analysis

**Figure 1:**
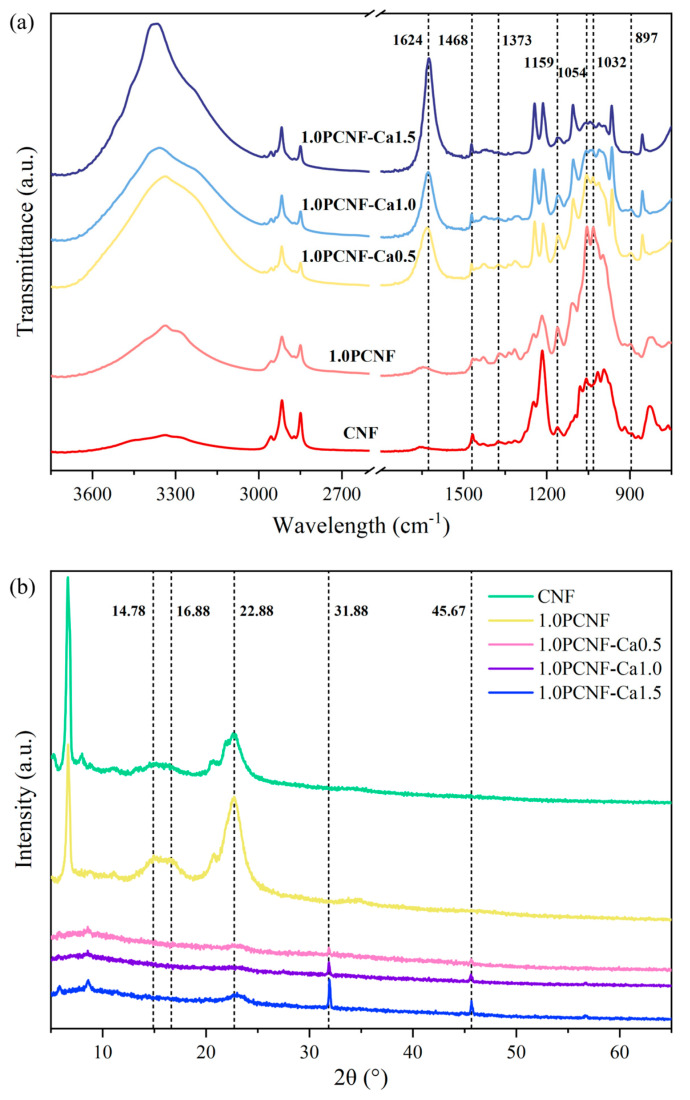
(**a**) FTIR spectra of CNF, 1.0PCNF, 1.0PCNF-Ca0.5, 1.0PCNF-Ca1.0, and 1.0PCNF-Ca1.5. (**b**) XRD curves of CNF, 1.0PCNF, 1.0PCNF-Ca0.5, 1.0PCNF-Ca1.0, and 1.0PCNF-Ca1.5.

As shown in Figure 1a, the porous flame-retardant material retained features similar to untreated cellulose, such as those corresponding to hydroxyl functional groups, and the peaks around 2900 cm^−1^, as well as those between 3000 and 2850 cm^−1^, corresponding to CH stretching. The characteristic absorption peaks of CNF at 1373 and 897 cm^−1^ correspond to the C-H bending vibration [17]. As the concentration of Ca^2+^ gradually increased, the peak gradually diminished, indicating that the C-H bending vibration progressively weakened. The peaks at 1032, 1159, 1373, and 1468 cm^−1^ are attributed to the C-O, C-O-C, C-H, and CH_2_ bending vibrations, respectively [18]. Additionally, the peak at 1054 cm^−1^ is also associated with C-O bending. The peak at 1054 cm^−1^ exhibited a red shift with the increase in Ca^2+^ content, indicating the occurrence of chemical interactions. The peak at 1032 cm^−1^ corresponds to O-H stretching and CH_2_ shear vibrations [19]. Notably, 1.0PCNF-Ca0.5, 1.0PCNF-Ca1.0, and 1.0PCNF-Ca1.5 exhibited characteristic absorption peaks at 1624 cm^−1^. The shift of the characteristic peak at 1624 cm^−1^ indicates the interaction between Ca^2+^ and the carboxyl and phosphate groups in PCNF, suggesting that Ca^2+^ was successfully deposited on the cellulose surface. The FTIR results indicate that CNF was transformed into PCNF through a phosphorylation process [20]. Furthermore, PCNF successfully interacted with Ca^2+^ via ionic bonds, resulting in the formation of a novel flame-retardant material.

### 2.3. Crystalline Structure

The curve in Figure 1b illustrates the typical cellulose structure. PCNF and CNF exhibited distinct crystalline peaks at 14.78°, 16.88°, and 22.88°, while 1.0PCNF-Ca 0.5, 1.0PCNF-Ca 1.0, and 1.0PCNF-Ca 1.5 presented characteristic peaks at 31.88° and 45.67°. The peak at 31.88° represents hydroxyapatite [21], while the peak at 45.67° corresponds to calcium pyrophosphate [22]. Following the cross-linking of Ca^2+^ with the phosphate group on phosphorylated cellulose, along with the deposition of Ca^2+^ on the cellulose surface [23], the hydrogen bond interactions in 1.0PCNF-Ca0.5, 1.0PCNF-Ca1.0, and 1.0PCNF-Ca1.5 were weakened, resulting in a decrease in crystallinity. The formation of hydrogen bonds is indicated by the O-H stretching vibration absorption peak located between approximately 3200 and 3600 cm^−1^. The blue shift of the spectral peak suggests that the addition of calcium ions reduced the hydrogen bond interactions within the aerogel [24]. Consequently, the peak values at 14.78° and 16.88° diminished sharply, and the peak at 22.88° also exhibited a reduction in intensity [25].

### 2.4. Morphological Observation

**Figure 2:**
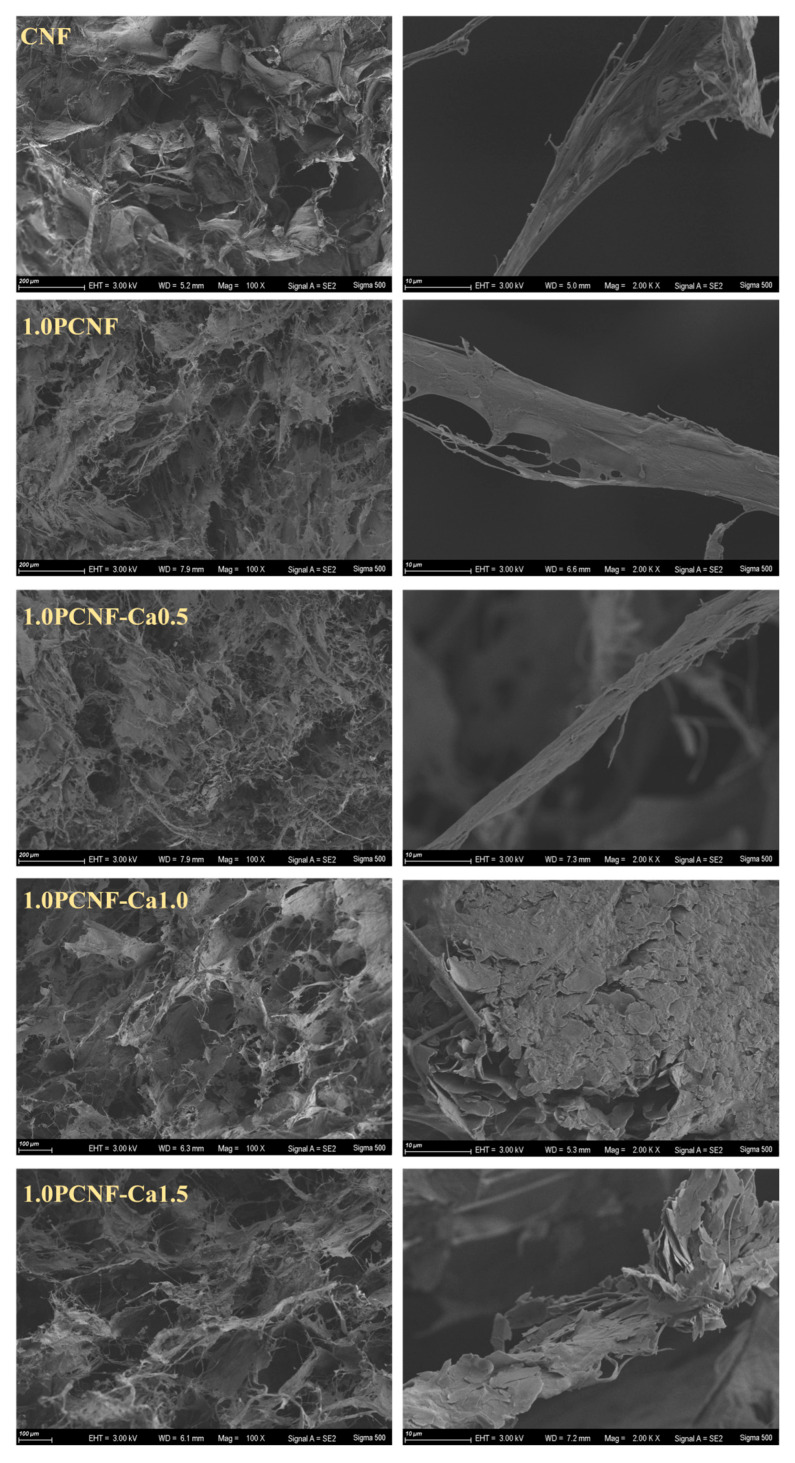
SEM images of CNF, 1.0PCNF, 1.0PCNF-Ca0.5, 1.0PCNF-Ca1.0, and 1.0PCNF-Ca1.5.

As shown in Figure 2, further morphological analysis of cellulose revealed that CNF, 1.0PCNF, 1.0PCNF-Ca0.5, 1.0PCNF-Ca1.0, and 1.0PCNF-Ca1.5 consisted of porous networks composed of microfibers. Notably, 1.0PCNF-Ca0.5, 1.0PCNF-Ca1.0, and 1.0PCNF-Ca1.5 exhibited tighter connections and greater strength due to cross-linking and the deposition of calcium ions with phosphorylated cellulose. In comparison to 1.0PCNF-Ca1.0, the microfibers of 1.0PCNF-Ca0.5 were excessively fine, while those of 1.0PCNF-Ca1.5 were fragmented. In conclusion, the morphology of 1.0PCNF-Ca1.0 is superior [26].

### 2.5. Flame-Retardant Performance

**Figure 3:**
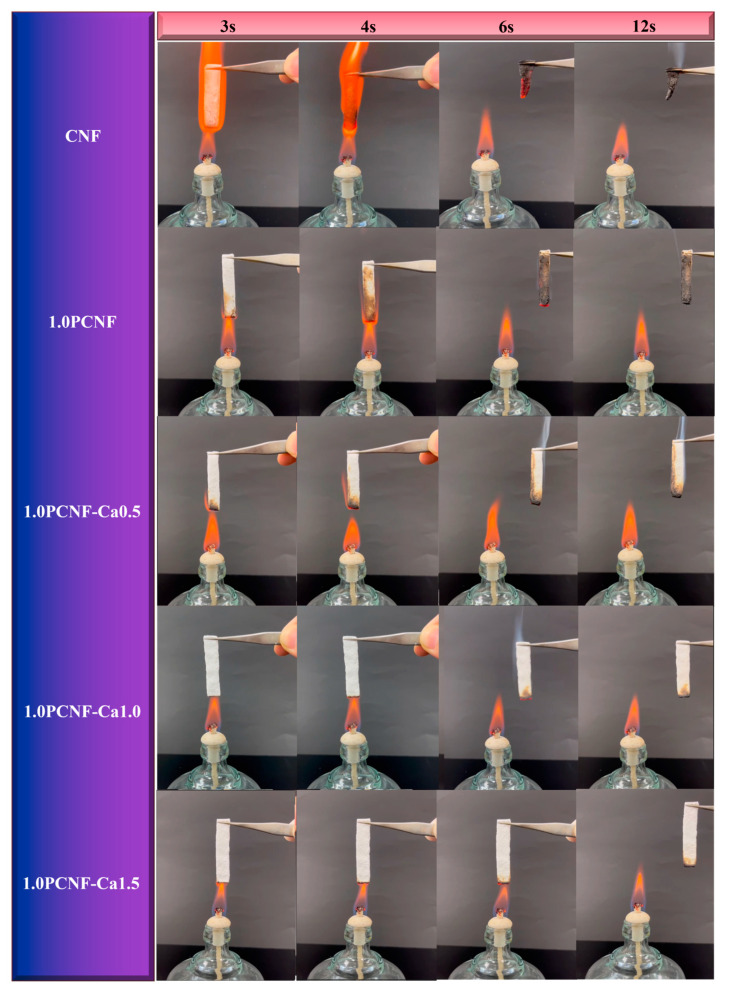
Flame-retardant test for CNF, 1.0PCNF, 1.0PCNF-Ca 0.5, 1.0PCNF-Ca1.0, and 1.0PCNF-Ca 1.5.

The results of the vertical combustion tests conducted on the prepared porous flame-retardant materials are presented in Figure 3. CNF did not undergo any modification treatment and was not dried before testing. It was observed that CNF exhibited no flame-retardant effect [27]. In fact, CNF ignited readily, with flames spreading rapidly in a vertical direction until the entire sample was consumed, resulting in the formation of a carbon residue. In contrast, the flammability of PCNF was significantly diminished, and small bubbles could be observed on its surface post-combustion. This phenomenon may be attributed to the formation of a protective carbon layer by the phosphorylated groups [28]. As illustrated in Figure 3, when the concentration of PCNF remained constant, the flame-retardant performance of the material improved with an increase in the concentration of Ca^2+^. Conversely, the PCNF-Ca composite flame-retardant material exhibited self-extinguishing behavior. These findings indicate that Ca^2+^ plays a significant role in enhancing flame-retardant performance [29].

**Figure 4:**
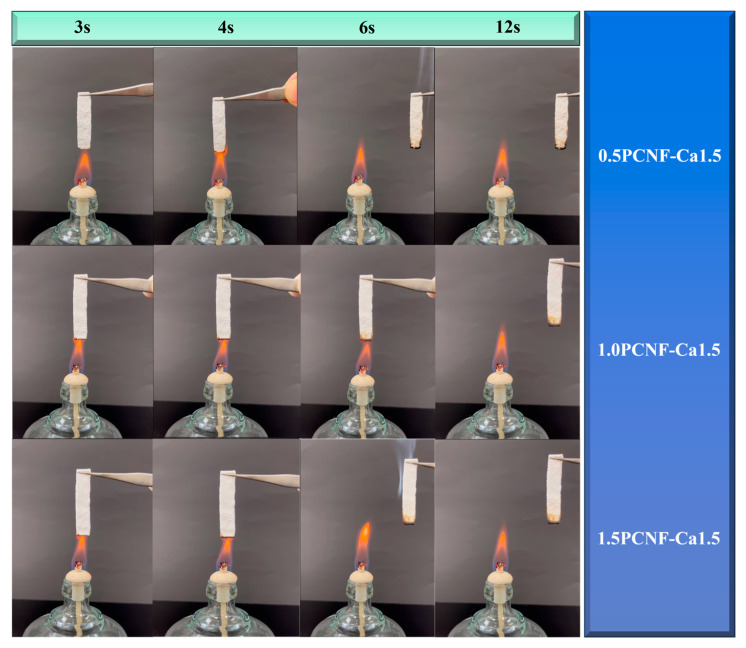
Flame-retardant test for 0.5PCNF-Ca1.5, 1.0PCNF-Ca1.5, and 1.5PCNF-Ca1.5.

Figure 4 further demonstrates that, with a constant concentration of Ca^2+^, the flame resistance of the material increased as the concentration of PCNF rose. The underlying mechanism may involve the excellent flame-retardant properties of calcium salts and the strong cross-linking effect of Ca^2+^ on cellulose [30]. This cross-linking led to a more compact cellulose network structure, reducing the oxygen content within the aerogel and thereby contributing to its flame-retardant effect. The UL-94 test results for PCNF reached a V-1 grade, while the UL-94 combustion test results for 1.0PCNF-Ca0.5, 1.0PCNF-Ca1.0, 1.0PCNF-Ca1.5, 0.5PCNF-Ca1.5, and 1.5PCNF-Ca1.5 achieved a V-0 grade [31]. Among these, the flame-retardant effects of 0.5PCNF-Ca1.5, 1.0PCNF-Ca1.5, and 1.5PCNF-Ca1.5 were found to be the most effective. The formation of the carbon layer created a physical barrier for the cellulose fibers, which reduced the transfer of oxygen and heat, thereby functioning as a flame retardant [32].

### 2.6. Mechanical Property Analysis

**Figure 5:**
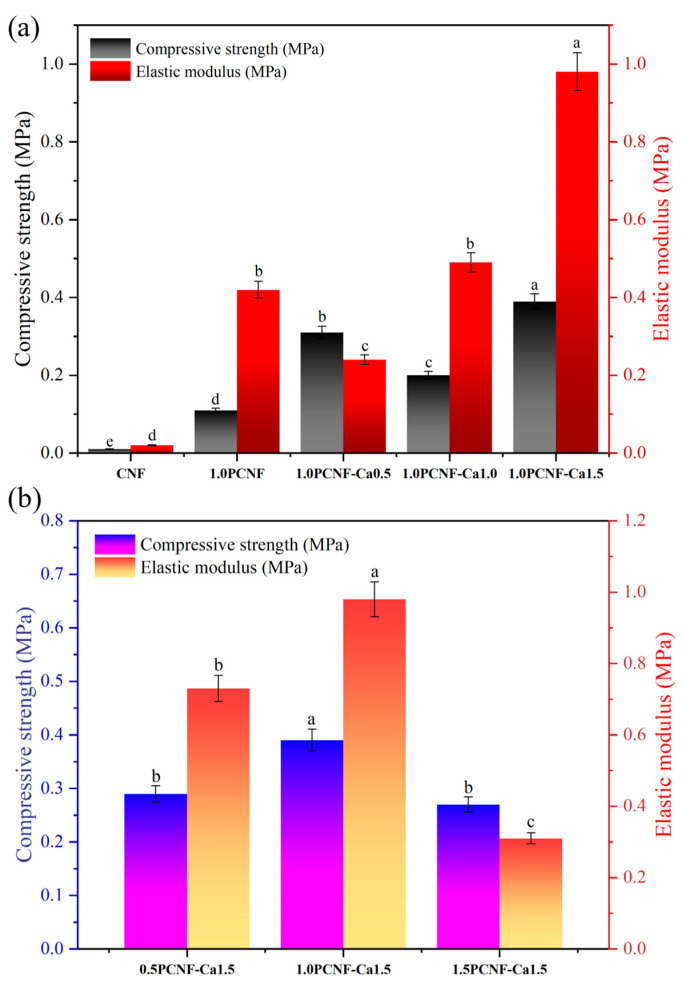
(**a**) Mechanical compression test of CNF, 1.0PCNF, 1.0PCNF-Ca0.5, 1.0PCNF-Ca1.0, and 1.0PCNF-Ca1.5. Letters a–e indicate significant differences between groups (*p* < 0.05). (**b**) Mechanical compression test of 0.5PCNF-Ca1.5, 1.0PCNF-Ca1.5, and 1.5PCNF-Ca1.5. Letters a–c indicate significant differences between groups (*p* < 0.05).

As illustrated in Figure 5a, when the concentration of PCNF remained constant, the mechanical properties generally improved with increasing concentrations of calcium ions [33]. This suggests that calcium ions facilitate the cross-linking of cellulose, resulting in a denser cellulose mesh structure and enhanced mechanical properties [34].

Conversely, as depicted in Figure 5b, when the amount of calcium ions was held constant, an increase in the PCNF concentration led to an initial enhancement in the mechanical properties of the material, followed by a decline [35]. This indicates that the optimal mechanical properties of the aerogel, comprising calcium ions and PCNF, were achieved only at a specific additive ratio. Overall, the combination of PCNF at a concentration of 1.0 and calcium ions at 1.5 yielded the best mechanical properties [36].

### 2.7. Thermal Stability

**Figure 6:**
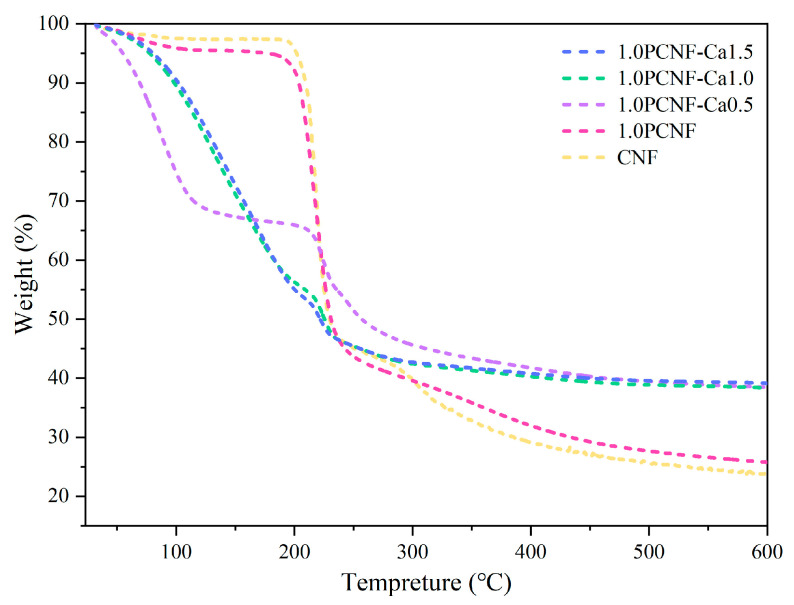
TG curves of CNF, 1.0PCNF, 1.0PCNF-Ca0.5, 1.0PCNF-Ca1.0, and 1.0PCNF-Ca1.5.

As shown in Figure 6, the peak degradation temperature of PCNF and CNF was 218.55 °C. Initially, the loss associated with the addition of calcium ions exceeded that of the samples without Ca^2+^. The inclusion of Ca^2+^ appeared to gradually reduce the thermal stability of cellulose [37], which may be related to the accelerated thermal cleavage of cellulose by the addition of Ca^2+^ [38]. This phenomenon may be attributed to the ability of Ca^2+^ to enhance heat conduction, thereby enabling cellulose to reach the degradation temperature more rapidly. However, as the temperature continued to rise, the final loss of the sample containing calcium ions was significantly lower than that of the sample without them [39]. The functionalization of phosphorylated cross-linked Ca^2+^ enhanced the thermal stability of CNF and served as an effective flame-retardant component. During the degradation process, a carbon layer formed on the cellulose, which improved the stability of the coating at elevated temperatures [40]. The primary benefit of carbonization was its ability to disrupt the contact between the aerogel and oxygen, thereby enhancing thermal stability. In conclusion, 1.0PCNF-Ca1.5 exhibited the lowest heat requirement for degradation [41].

### 2.8. Mechanism Analysis

**Figure 7:**
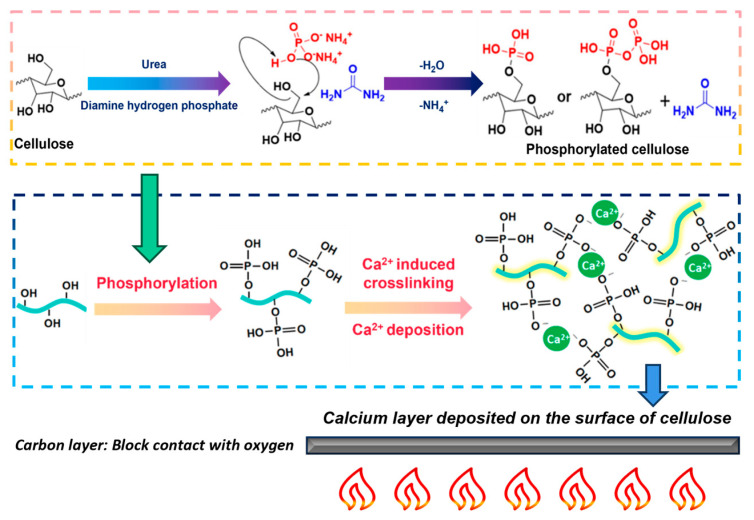
Schematic of chemical modification of porous flame-retardant materials.

The principles underlying the chemical modification are illustrated in Figure 7. The hydroxyl groups of cellulose were combined through covalent (especially phosphoester) linkages. Under heating conditions, diammonium phosphate generated ammonia gas, water vapor, and substances containing phosphate ions [42]. Freeze-drying removed H_2_O and NH_4_^+^, resulting in the formation of phosphorylated cellulose, which reduced the number of free hydroxyl groups and enhanced both the mechanical and flame-retardant properties [43]. Phosphorylation reactions conferred flame retardancy to the cellulose at high temperatures. However, they also increased carbonization reactions. Subsequently, calcium ions contributed to the preparation of the material through self-assembly and surface deposition effects [44], thereby achieving more effective flame retardancy. The specific effects can be observed in the video provided in the Supplementary Materials. During combustion, a carbonized layer formed on the surface of the material, which minimized the introduction of oxygen and thereby improved the flame-retardant effect [45]. As the temperature rose, the rate of carbon oxidation accelerated, while the supply of oxygen gradually became insufficient. The rate of oxygen transport within the boundary layer played a critical role in controlling the erosion rate [46].

## 3. Conclusions

In this study, vertical combustion tests, infrared characterization, and scanning electron microscopy were employed to evaluate the mechanical properties and flame-retardant performance of cellulose aerogels. The 1.0PCNF-Ca1.5 sample exhibited the highest performance due to its elevated cross-linking density and optimal mechanical properties. The results from the UL-94 test indicated a V-0 rating, demonstrating a flame-retardant efficiency of 90.6%. The flame-retardant mechanism of the high-performance cellulose-based materials, prepared through phosphorylation coupled with calcium ion coordination cross-linking, relied on the phosphorylation process to impart flame-retardant properties, which were subsequently enhanced by the addition of calcium ion cross-linking to the cellulose. Calcium salts act as intrinsic flame-retardant materials, thereby augmenting the overall flame-retardant performance of the aerogel. Furthermore, calcium ion cross-linking effectively increased the density of the cellulose network, reduced the oxygen content within the material, and enhanced the overall flame-retardant effect. This cross-linking process contributed to a more compact cellulose network, thereby improving the mechanical properties of the cellulose-based flame-retardant material. The findings of this study address the challenges associated with the development and utilization of cellulose due to its inherent properties, offering innovative strategies for fire prevention and control. The resulting porous flame-retardant material exhibited advantages such as non-toxicity, biodegradability, recyclability, and environmental friendliness, aligning with the principles of green development. Plastic products utilized in construction materials exhibit resistance to degradation in natural environments. Conversely, building materials derived from cellulose demonstrate the ability to undergo natural degradation. This positions it as an ideal lightweight flame-retardant material with the potential for large-scale applications as the next generation of innovative green building materials.

## 4. Materials and Methods

### 4.1. Materials and Chemicals

Lignocellulosic paper pulp was procured from Guilin Qihong Technology Co., Ltd., Guilin, China, while ammonium dihydrogen phosphate, urea, anhydrous calcium chloride, and sodium dodecyl sulfate (which helps to deposit Ca^2+^ onto cellulose) [47] were sourced from Sinopharm Chemical Reagent Co., Ltd. (Shanghai, China). All reagents were of analytical grade and were ready for use without the need for further processing.

### 4.2. Preparation of Cellulose Nanofibers (CNFs) and Phosphorylated Cellulose Nanofibers (PCNFs)

Initially, 20.0 g of pulp was soaked in deionized water and stirred with a mixer for 5 min to obtain the cellulose nanofiber (CNF) control group. Subsequently, the same 20.0 g of pulp was soaked in deionized water and stirred for an additional 5 min. This was followed by mixing with 74.0 g of urea and 18.0 g of ammonium dihydrogen phosphate. The resulting mixture was stirred for another 5 min to ensure homogeneity. Both solutions were then heated at 80 °C for 30 min. After heating, the two types of samples were dried at 105 °C until a constant weight was achieved, followed by curing in an oven at 150 °C for 30 min. After dialysis, vacuum filtration was employed to remove residual reagents adhering to the surfaces until the conductivity of the filtrate dropped below 10 μS cm^−1^. Finally, the phosphorylated pulp sample was dispersed in deionized water at a concentration of 1 wt% to prepare solutions of cellulose pulp (CNF) and phosphorylated cellulose pulp (PCNF).

### 4.3. Preparation of Porous Flame-Retardant Aerogel Materials

The phosphorylated cellulose solution was utilized to prepare cellulose aerogels at total solid concentrations of 0.5, 1, and 1.5 wt%, designated as 0.5CNF, 1.0CNF, and 1.5CNF, respectively. Subsequently, samples with concentrations of 0.5, 1.0, and 1.5 wt% were prepared by adding 0, 0.75, 1.50, and 2.25 g of calcium chloride, respectively. These samples were named 0.5PCNF, 0.5PCNF-Ca0.5, 0.5PCNF-Ca1.0, and 0.5PCNF-Ca1.5. Similarly, by adding the same amount of calcium chloride, samples with a concentration of 1 wt% were prepared and named 1.0PCNF, 1.0PCNF-Ca0.5, 1.0PCNF-Ca1.0, and 1.0PCNF-Ca1.5, respectively. Finally, samples with a concentration of 1.5 wt% were prepared using the same calcium chloride additive and named 1.5PCNF, 1.5PCNF-Ca0.5, 1.5PCNF-Ca1.0, and 1.5PCNF-Ca1.5, respectively. All of the aforementioned samples were homogenized using a high-speed homogenizer operated at 12,000 rpm for 5 min. The RCD-1A high-speed homogenizer model was used, which was purchased from Ltd. The resulting mixtures were then poured onto culture plates, with each plate containing an average mass of 25.0 g from a total of three plates. The sample was stored in a −80 °C refrigerator for 30 min and subsequently vacuum freeze-dried using an LGJ-18 freeze dryer, purchased from Beijing Songyuan Huaxing Technology Development Co., Ltd., Beijing, China, for a duration of 48 h. The chamber pressure was 10–30 Pa. Once the temperature inside the chamber reached room temperature, the sample was removed.

### 4.4. Density Test

After drying the sample to a constant weight, the mass of the dried sample was measured using a laboratory balance with an accuracy of 0.01 g. A ruler was employed to measure the length, width, and height of the samples to calculate their volume. To obtain the density of the sample, Formula (1) was used for density. Each sample was measured five times, and the average value was recorded [48].

(1) $$Density\left(\frac{g}{{cm}^{3}}\right)=\frac{Mass\left(g\right)}{Volume\left({cm}^{3}\right)}$$

### 4.5. Flame-Retardant Test

UL-94 vertical combustion: According to the ASTM D3801-19 standard method [49], the evaluation of flame-retardant performance in materials exposed to flame was categorized into multiple levels, including V-0, V-1, V-2, HB, and others. Among these, V-0 represents the highest level of performance, necessitating that the material extinguishes the flame within 10 s after undergoing two combustion tests, with no droplets igniting the cotton pad during the process. At this point, the burning time was recorded, and photographs were taken to document the combustion process [50].

### 4.6. Fourier Transform Infrared (FTIR)

The samples were analyzed using an FTIR spectrometer (470-Nexus, Nicolet, Madison, WI, USA) in absorbance mode across a range of 750 to 3750 cm^−1^. Sixteen scans were conducted at a temperature of 25 °C, achieving a resolution of 4 cm^−1^ [51].

### 4.7. X-Ray Diffraction (XRD)

The X-ray diffractometer (PANalytical X’pert Pro MRD, Amsterdam, The Netherlands) utilized for the analysis operated at a current of 40 mA and a voltage of 40 mV to examine the diffraction patterns. Scans were performed in the 2θ range of 5–65°. To determine the crystallinity index, the conventional Segal method was employed [52].

### 4.8. Thermogravimetric Analysis (TGA)

The thermal stability of the samples was assessed utilizing a thermogravimetric analyzer (TG 209F3 Tarsus, NETZSCH, Selb, Germany). During this evaluation process, the samples were subjected to a controlled heating procedure, which involved increasing the temperature from an initial value of 30 °C up to a maximum of 800 °C. This temperature change was conducted at a consistent ramp rate of 10 °C per minute. Furthermore, the analysis was performed under a nitrogen flow maintained at 250 cm^3^/min to ensure an inert atmosphere throughout the thermal testing [53].

### 4.9. Compression Test

Tests for uniaxial static compression were conducted in compliance with ASTM D1621 [54], which specifies the “Standard Test Method for Compressive Properties of Rigid Cellular Plastics”. The elastic modulus and compressive strength were evaluated from these experiments. Compressive strength was defined as the stress level at a strain of 10%, whereas toughness was derived as the integral of the stress–strain curve from 0% to 40% strain [55].

### 4.10. Scanning Electron Microscopy (SEM)

A high-resolution sputtering coater, model Cressington 208HR (Hertfordshire, UK), was utilized to apply a gold coating with a thickness ranging from 3 to 5 nm on the samples. Subsequently, the samples were examined morphologically using a Hitachi S-4800 field emission scanning electron microscope (Tokyo, Japan) [56].

### 4.11. Statistical Analysis

Statistical analyses were performed to identify significant differences between the results obtained using SPSS 26.0. Measurements in this study were performed in triplicate unless otherwise indicated.

## Figures and Tables

**Table 1:** Data on the diameter, thickness, quality, and density of the cellulose aerogel samples.

Samples	Diameter (mm)	Thickness (mm)	Weight (g)	Density (g/cm^3^)
CNF	53.870 ± 1.280	8.900 ± 0.880	0.390 ± 0.090	0.019 ± 0.001
0.5PCNF	54.000 ± 1.110	9.700 ± 0.970	0.200 ± 0.090	0.009 ± 0.003
1.0PCNF	54.400 ± 1.250	9.500 ± 0.450	0.290 ± 0.050	0.013 ± 0.002
1.5PCNF	54.270 ± 0.960	10.30 ± 0.890	0.520 ± 0.070	0.022 ± 0.003
0.5PCNF-Ca0.5	52.430 ± 0.910	7.470 ± 0.830	0.590 ± 0.040	0.037 ± 0.004
1.0PCNF-Ca0.5	52.570 ± 1.010	7.170 ± 0.850	0.670 ± 0.040	0.043 ± 0.003
1.5PCNF-Ca0.5	52.400 ± 0.980	8.800 ± 0.790	0.840 ± 0.060	0.044 ± 0.006
0.5PCNF-Ca1.0	51.670 ± 0.950	6.430 ± 0.970	0.880 ± 0.070	0.065 ± 0.005
1.0PCNF-Ca1.0	51.530 ± 0.860	6.230 ± 0.990	0.970 ± 0.050	0.075 ± 0.002
1.5PCNF-Ca1.0	51.570 ± 0.890	7.330 ± 0.730	1.100 ± 0.050	0.072 ± 0.001
0.5PCNF-Ca1.5	49.400 ± 0.940	5.770 ± 0.870	1.200 ± 0.060	0.103 ± 0.008
1.0PCNF-Ca1.5	50.030 ± 0.820	5.800 ± 0.570	1.310 ± 0.040	0.110 ± 0.001
1.5PCNF-Ca1.5	50.930 ± 1.130	7.000 ± 0.740	1.410 ± 0.080	0.099 ± 0.003

## Data Availability

All data are contained within the article.

## Supplementary Materials

The following supporting information can be downloaded at: https://www.mdpi.com/article/10.3390/gels11060408/s1, Video S1: CNF, Video S2: 1.0PCNF, Video S3: 1.0PCNF-Ca0.5, Video S4: 1.0PCNF-Ca1.0, Video S5: 1.0PCNF-Ca1.5.
